# Quality evaluation of guidelines for the diagnosis and treatment of radiation enteritis

**DOI:** 10.1186/s13014-023-02204-9

**Published:** 2023-01-20

**Authors:** Xiao-Feng Yang, Meng-Yao Zheng, Li-Ya An, Jin-Min Sun, Qian-Wen Hei, Yan-Hong Ji, Da-Li Sun, Hai-Yu He

**Affiliations:** 1 Department of Gastrointestinal Surgery, Second Affiliated Hospital of Kunming Medical University/Second Faculty of Clinical Medicine, Kunming Medical University, Kunming, 650101 China; 2 Department of Gastroenterology, Second Affiliated Hospital of Kunming Medical University/Second Faculty of Clinical Medicine, Kunming Medical University, Kunming, 650101 China

**Keywords:** Radiation enteritis, Treatment, Quality evaluation, Guidelines

## Abstract

**Objective:**

To systematically evaluate the guidelines for the diagnosis and treatment of radioactive enteritis, compare their differences and reasons and provide some reference for updating them.

**Methods:**

This study used guidelines related to radiation enteritis by searching a database. Four independent reviewers used the AGREE II evaluation tool to evaluate the quality of the included guidelines, collate their main recommendations, and analyze the highest evidence supporting the main recommendations.

**Results:**

Six diagnostic and therapeutic guidelines for radiation enteritis were included in this study, one of which, the American Society for Gastrointestinal Endoscopy guidelines, had an overall score of over 60%, which is worthy of clinical recommendation. In the diagnosis and treatment of radioactive rectal injury, the recommendations for hemorrhagic endoscopic treatment are mature and mainly include (I) argon plasma coagulation; (II) formalin treatment; (III) bipolar electrocoagulation; (IV) heater probe; (V) radiofrequency ablation; and (VI) cryoablation.

**Conclusion:**

The methodological quality of radioactive enteritis guidelines is unequal; even in the same guidelines, different domains have a large difference. For radioactive rectal damage diagnosis, a type of endoscopic treatment recommendation is more mature, but the overall diagnosis and treatment of radioactive enteritis still lacks high-quality research evidence.

**Supplementary Information:**

The online version contains supplementary material available at 10.1186/s13014-023-02204-9.

## Key point

- This article reviews the diagnosis and treatment guidelines for radiation enteritis in order to promote further update of the guidelines.

**Supplementary Information:**

The online version contains supplementary material available at 10.1186/s13014-023-02204-9.

## Introduction

Radiation enteritis refers to the intestinal radiation damage caused by radiotherapy in patients with pelvic malignancies such as bladder cancer, cervical cancer, endometrial cancer, ovarian cancer, prostate cancer and rectal cancer. According to the onset time, course and location of the disease, it can be divided into acute radiation enteritis, acute radiation proctitis, chronic radiation enteritis, and chronic radiation proctitis [1]. In the 2021 edition of the consensus of multidisciplinary experts on the diagnosis and treatment of radioactive rectal injury [2], radioactive rectal injury was first classified into the capillary dilatation type, mainly manifested as hematochezia; ulcer type, with rectal symptoms including anal distension anal pain, increased number of stools, urgent, mucous stool, tenesmus and fecal incontinence; stenosis type, which, according to the different degrees of stenosis, can manifest as lower abdominal pain, defecation difficulty, reduced defecation and fecal thinning, and small bowel obstruction symptoms; and mixed type, in which the symptoms are complex and varied. The number of new cases of malignant pelvic tumors in China in 2015 alone exceeded 500,000 [3]; more than 61% of patients with malignant pelvic tumors received pelvic radiation therapy, 75% of patients receiving pelvic radiation therapy developed acute radioactive rectal injury, and 5–20% developed chronic radioactive rectal injury [4]. The incidence of this disease may be seriously underestimated. (I) Gamid et al. [5] reported that 81% of patients who received pelvic radiotherapy experienced gastrointestinal symptoms, and only 55% of patients sought help from doctors. (II) Patients with chronic radioactive rectal injury have prolonged and repeated symptoms and are prone to late serious complications, such as massive gastrointestinal bleeding, perforation, obstruction, and intestinal fistula, which seriously affect the quality of life of patients and bring great challenges to the diagnosis and treatment of the disease. (III) There are few clinically relevant studies and few and poor-quality guidelines, and many therapies lack safety testing. The existence of these conditions makes it particularly important to formulate high-quality guidelines for the diagnosis and treatment of radioactive enteritis.

Obviously, this situation has also been considered important by different experts in various countries. In recent years, many guidelines on how to treat radiation enteritis have been formulated [1, 2, 6–9], but the quality of these guidelines and recommendations are irregular, making it inconvenient for clinicians to apply them. The purpose of this study was to find a more appropriate program for clinicians to apply by sorting out and evaluating the quality of recommendations of various guidelines and to provide a basis for further development of higher quality guidelines.

## Methods

### Study design

This study comprehensively evaluated and analyzed the guidelines for the diagnosis and treatment of radioactive enteritis by using the AGREE tool. This study followed the Preferred Reporting Items for Systematic Reviews and Meta-analysis Protocols (PRISMA-P) statement [10].

### Retrieval strategies

In this study, the OVID, Web of Science, ScienceDirect, PubMed, CBM, CNKI and other databases were searched, and at the same time, the official website of the Gastrointestinal Diseases Association, American Gastroenterology Association (AGA) and related website of the guidelines of Yimai Tong were searched without language restrictions. Considering the time limit of evidence, we only included guidelines from 2011 to 2021. Considering the existence of multiple translations of the word, we used variations of the guidelines for radioactive enteritis to make the retrieval comprehensive using the following search terms:, “radiation enteritis”, “radiation enterocolitis”, “guide”, “guideline”, manual”, “guidance”, “recommendation”, and “consensus”, which were used in our study. At the same time, the references of the included guidelines related to radioactive enteritis were manually retrieved in this study.

### Selection of guidelines

A series of inclusion and exclusion criteria were established in the selection of the literature in this study. The inclusion criteria were as follows: (I) the study population was patients with radiation enteritis; (II) the full text was available online; and (III) the guide was the latest version. The exclusion criteria for the guidelines were as follows: (I) guidelines that were not closely related to radioactive damage; (II) duplicate reports; (III) unavailable interpretation of the guidelines; and (IV) full texts were not available. The literature was selected by two authors independently according to the above inclusion and exclusion criteria using EndNote (X9). The guidelines with high relevance to this study were selected by reading their abstracts and titles. When two authors had disputes over the selection of the guidelines, the third author participated in the selection and further discussed the selection of the guidelines. At the same time, basic information such as the title of the guide, the year of publication, the first author and the main content of the guide was extracted.

### Quality evaluation of the guidelines

We evaluated the quality of the selected guidelines by using the latest version of the AGREE II Tool (2017 version) [11]. The AGREE II tool is a tried and tested guideline quality evaluation tool designed to provide a framework for measuring and quantifying the quality of guidelines. AGREE II defines the quality of the guidelines with full consideration of potential bias in the development of the guidelines and confidence in the internal and external authenticity of the guideline recommendations and feasibility of implementation [11]. The AGREE II tool includes 23 items in 6 areas: Area 1: Scope and purpose, which relate to the overall objectives of the guidelines, specific health problems and target groups (Items 1–3), and areas such as implementing specific clinical problems or health themes and clarifying major recommendations; Area 2: Participants, mainly including the professional staff of the formulation group and the positions of their units, the users of the guidelines, whether public opinions are considered in the formulation process, etc. (Items 4–6); Area 3: Rigor of formulation, including the process of collecting, screening, and voting on opinions (Items 7–14); Area 4: Clarity of expression, clarity of opinions, and identification of users and conditions (Items 15–17); Area 5: applicability, including suitability and hindrance factors in use and whether direct audit indicators are available for clinical application (Items 18–21); and Area 6: Editorial independence, ensuring that the interests of each fund panel member do not bias the results (Items 22–23). Each area was independently evaluated by four reviewers (Xiao-feng Yang, Yan-Hong Ji, Jin-min Sun, and Qian-wen Hei). Each item was scored on a 7-point scale: 1 point meant strongly disagree, 7 points meant strongly agree that the item was not mentioned at all, and 1 point was given. If the content mentioned in the article did not completely conform to the item, the score ranged from 2 to 6. When there was a difference of more than 3 points between the scores of four reviewers for the same item, the four reviewers discussed and adjusted the score again. After all scores were combined and counted by a reviewer, the score of each field was calculated using the formula (score obtained − minimum possible score)/(maximum possible score − minimum possible score) × 100%. After the results were obtained in the previous step and the reviewers analyzed them, the included literature was divided into three categories: recommended (R > 60%), recommended with modifications (RM 30–60%), and not recommended (NR < 30%).

### Statistical analysis

In this study, standardized scores for each domain were calculated using descriptive statistical analysis, expressed as percentages and presented in tabular form as averages and ranges. We used two-way analysis of variance to calculate the intragroup correlation coefficients (ICCs) to test whether the scores of the four evaluators were consistent. An ICC value greater than 80% was considered to indicate good agreement among the four evaluators. The statistical software used in this study was SPSS Version 26.0 (SPSS Inc., Chicago, IL, USA).

### Guidelines for the evaluation of items and evidence related to radiation enteritis diagnosis and treatment

We consulted guidelines with a relatively high AGREE II score to extract and analyze significant recommendations related to the treatment of radioactive enteritis to further obtain and analyze the highest level of evidence supporting these recommendations and the highest evidence currently available in the search database. The level of recommendation was determined by reclassifying this evidence using the Oxford Centre for Evidence-Based Medicine (OCEBM) grading system (Additional file1: Table S1) [11].

## Results

### Features of included guidelines

**Fig. 1:**
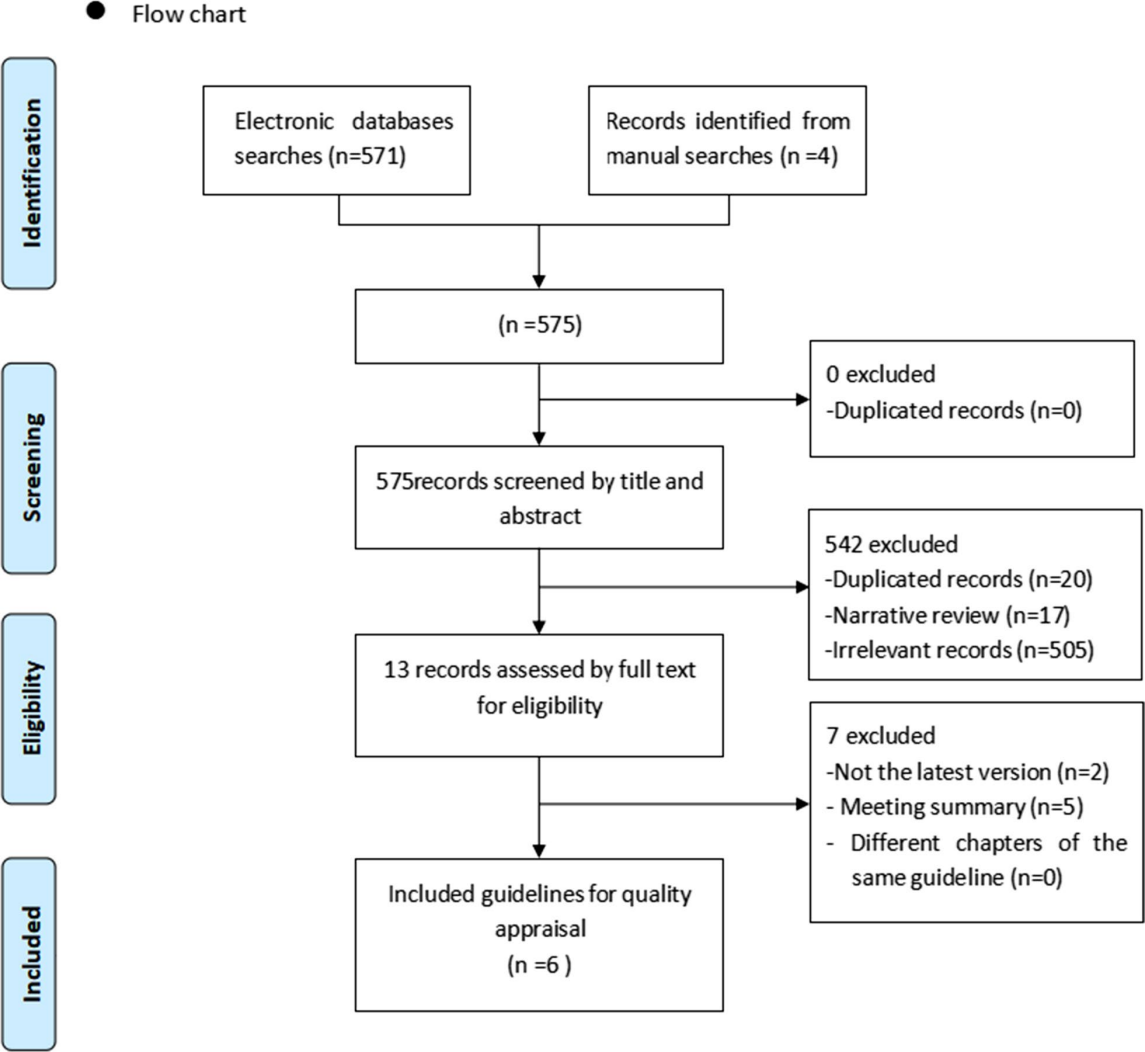
Flow chart of study selection

A total of 575 records were initially searched, and 6 guidelines meeting the inclusion criteria were screened out through title content (Fig. 1). The features of the 6 guidelines included in this study are shown in Table 1.

**Table 1:** Characteristics of the identified guidelines for the diagnosis of pancreatic cancer

Title	Author/organization	Short name	Development organization	Version	Topic	Funding
The American Society of Colon and Rectal Surgeons Clinical Practice Guidelines for the Treatment ofChronic Radiation Proctitis [6]	Paquette IM, et al	Pa (1)	The Clinical Practice Guidelines Committee of the American Society of Colon and Rectal Surgeons	Original version	The treatment of chronic radiation proctitis	None
Practice guidance on the management of acute and chronic gastrointestinal problems arising as a result of treatment for cancer [7]	Andreyev HJ et al	An (2)	Pelvic Radiation Disease and GI Unit, Royal Marsden NHS Foundation Trust, London and Sutton, UK	Original version	Late management of cancer treatment and comparison of various treatment methods	None
Chinese expert consensus on multidisciplinary diagnosis and treatment of radiation rectal injury (2021 edition) [2]	Shuqun C et al	Sc (3)	Surgeon branch of Chinese medical doctors association	Adapted version	Diagnosis and treatment of radiation proctitis	None
Radiation-induced small bowel disease						
Latest developments and clinical guidance [8]	Stacey R et al	St (4)	Not mentioned	Original version	Management of radiation-induced intestinal diseases	None
ASGE guideline on the role of endoscopy for bleeding from chronic radiation proctopathy [9]	Lee JK et al	Le (5)	American Society for Gastrointestinal Endoscopy(ASGE)	Adapted version	The role of endoscopy for bleeding from chronic radiation proctopathy	The National Cancer Institute/National Institutes of Health
Expert Consensus on Surgical Management of Chronic Radioactive Intestinal Injury (2019 Edition) [1]	CMA	CM (6)	Department of Gastrointestinal Surgery, Chinese Surgical Society	Original version	Surgical treatment of radioactive intestinal injury	None

A total of six guidelines published between 2011 and 2021 were included, including those put forward by the American Society of Colorectal Surgery, American Society of Gastrointestinal Endoscopy, Chinese Society of Surgery, Gastrointestinal Surgery Group, Chinese Medical Doctors Association, Surgeons Branch and other organizations. Four of them were original versions [1, 6–8], and two were updated versions [2, 9]. The guidelines focused on the treatment of radiation enteritis, such as the later management of cancer treatment, intestinal management of radiation enteritis, and endoscopic treatment of hemorrhagic radiation enteritis.

### Quality evaluation of the guides

Four reviewers used the AGREE II tool to score, and the scoring results are presented in Table 2, in which the scope and purpose of domain 1 were 48.13% (45.8–65.3%), and the clarity of expression of domain 2 was 45.38% (26.4–54.2%). In domain 3, 43.13% of participants (range 13.0–65.1%); the preciseness of domain 4 was 68.75% (range 45.8–83.3%); the mean value of domain 5 application was 23.6% (0–56.3%); and the median value of editorial independence in domain 6 was 58.7% (range 0–87.5%). Based on these scores, we decided whether to recommend the use of these guidelines. The scores of each guide are presented in Table 2. Only one guide [9] was rated above 60%, meeting the criteria for recommended use. The remaining six were rated between 30 and 60% and could be recommended after improvement. The scores of each guide are presented in Table 2. The evaluation of the guidelines for radioactive enteritis was completed by four reviewers, and the ICCs were all greater than 0.8, indicating a relatively high consistency of the evaluations among the four reviewers.

**Table 2:** AGREE II domain score and ICCs of the included guidelines

Guideline	Scope and purpose (%)	Stakeholder involvement (%)	Rigor of development (%)	Clarity and presentation (%)	Applicability (%)	Editorial independence (%)	Overall assessment (%)	
Paquette IM et al. 2018 (1)	45.8	54.2	52.1	61.1	0	64.6	41.2	RM
Andreyev HJ et al.,2012 (2)	62.5	54.2	33.3	61.1	18.8	50.0	41.5	RM
Shuqun C et al	58.3	45.8	51.0	83.3	35.4	87.5	56.0	RM
Stacey R et al. 2014 (4)	56.9	26.4	13.0	45.8	12.5	85.4	33.2	RM
Lee JK et al. 2019 (5)	56.9	40.3	65.1	79.2	56.3	64.6	60.5	R
CMA2019 (6)	65.3	51.4	44.3	82.0	18.8	0	40.6	RM
ICC	0.835	0.984	0.972	0.868	0.926	0.963		
Median score	48.13	45.38	43.13	68.75	23.6	58.7		
Range	45.8–65.3	26.4–54.2	13.0–65.1	45.8–83.3	0–56.3	0–87.5		

### Radiation enteritis diagnosis and treatment guidelines for main recommendations and the best evidence to date

To further analyze and compare the main recommendations of various guidelines, we took the guide [9] with the highest score as the standard reference to summarize the important recommendations related to the diagnosis and treatment of radioactive enteritis, including argon plasma coagulation, formalin treatment, bipolar electrocoagulation, heater probe, radiofrequency ablation and cryoablation. At the same time, the best evidence provided by each guide for making recommendations was determined, and the evidence provided by the guides was graded and recommended by using the evidence grading system of the Oxford Centre for Evidence-Based Medicine (OCEBM) (Table 3).

**Table 3:** Key recommendations and best evidence for the diagnosis and treatment of radiation proctitis and pelvic radiation disease in the included guidelines

The key recommendations	The best evidence to support the recommendations at present	Strength of recommendation	Quality of evidence	Pa (1)	An (2)	Sc (3)	St (4)	CM (6)
1. Argon plasma coagulation	A meta-analysis of 957 patients included in 33 studies showed that the overall clinical success rate of APC in treating hematochezia was 87% [2, 9]	B	2a	•	–	•	•	–
2. Formalin	A meta-analysis of 6 studies of 182 patients confirmed the efficacy of topical formalin [6]	B	2a	•	#	•	–	–
3. Bipolar electrocoagulation	Four studies, three randomized controlled trials and one case report were included, with a total of 96 patients and an overall response rate of 88% [13, 19–21]	A	1b	•	–	•	–	–
4. Heater probe	A randomized controlled trial with nine patients with a 67% clinical response rate [9]	B	2b	–	–	•	–	–
5. Radiofrequency ablation (RFA)	Three cases were reported with a total of 66 patients, with an overall response rate of 97.7% [9]	C	4	–	–	–	–	–
6. Cryoablation	A case series of 7 patients with rectal bleeding from chronic radiation proctopathy that was refractory to other endoscopic therapy [27]	C	4	–	–	–	–	–

*OCEBM* Oxford Centre for Evidence-Based Medicine

^•^ Indicates a definite recommendation; ^#^ indicates a mention; –indicates not mentioned. *, strength of recommendation and quality of evidence were assessed by using the OCEBM standard

## Discussion

### Principal findings

This study found that guidelines for the treatment of radioactive enteritis were of mixed quality. The treatment methods mentioned in different radiation enteritis guidelines vary widely. The main causes for this include radioactive enteritis studied by RCT experiments, the unknown treatment safety of various studies, the different evidence classification systems, irregular guideline rating systems, and the difficulty in diagnosing radioactive enteritis or misdiagnosing it as other inflammatory drug intestinal infectious diseases, which can delay treatment. A diagnosis is made even after complications such as perforation and bleeding of an obstructed intestinal fistula occur [12]. Moreover, the treatment methods mentioned in the clinical guidelines are not comprehensive, and more high-quality guidelines, such as those formulated by the American Society for Gastrointestinal Endoscopy (ASGE), are needed to guide clinical work [9]. The recommendations included in the guidelines varied widely; therefore, we further analyzed the consistency and controversy between current recommendations and the corresponding evidence for the management of radioactive enteritis.

### Quality evaluation of guidelines by AGREE II

According to the AGREE II tool, the range and purpose of guidelines and the scores of rigor and application formulated by stakeholders are relatively low, with mean values of 48.13% and 45.38%, 43.13% and 23.60%, respectively, and the scores of other domains were more than 50%. The reason for the lower average score of domain 1 was that the guidelines did not clearly indicate the application population and did not clearly define the age and sex of patients. The reason for the low mean value of stakeholder score in area 2 is that most guidelines ignored the consideration of public interests and failed to specify the users of the guidelines. It is believed that more consideration in these two aspects will be helpful for the formulation of higher quality guidelines. The lack of precision in area 3 is mainly because most of the guidelines did not mention the personnel who participated in the review of the guidelines. If a third party is invited to review the quality of the guidelines during their formulation, it is believed that the quality of the guidelines will be better. The applied score of area 5 was low; the main reason is that there was no valid reference to the application of promotion and hindrance factors, which is easy to find. If in the future the guidelines improve in these areas, there will be great progress in the diagnosis and treatment of radioactive enteritis.

### Problems and possible causes of the recommendations and supporting evidence in the guidelines for the diagnosis and treatment of radioactive enteritis

It is particularly important to use uniform evidence grading and evaluation criteria when formulating guidelines. Because different guidelines adopt different evidence evaluation systems, which is not conducive to readers' comparison, OCEBM was used in this study to re-evaluate and grade the evidence. The fact that most of the supporting evidence mentions safety uncertainty is also troubling. This reflects the lack of scientific investment in this disease and the need for more high-quality RCTs, which is a major obstacle to the development of high-quality guidelines.

#### Argon plasma coagulation (APC) (recommendation strength: B; level of evidence: 2A) [2, 6, 9, 12–16]

Four guidelines supported the use of argon plasma coagulation [2, 6, 8, 9], and two guidelines [1, 7] did not mention it. Two guidelines [2, 9] recommended argon plasma coagulation and referred to the meta-analysis of 33 studies involving 957 patients with an overall success rate of 87% [9]. The sample size of two case-report studies that mentioned the efficacy of argon-plasma coagulation versus formalin is 22 [16] and 30 [17], respectively. The former concluded that the efficacy of APC was superior to local formalin spot coating, while the latter concluded that the efficacy of APC was equivalent. In a randomized controlled trial of 122 patients [12], the clinical severity score decreased from 2 to 0 after 16 weeks, supporting the effectiveness of APC treatment. Guideline [6] referred to seven studies, 430 cases in total, that referred to the efficacy and safety of APC and therefore clearly support the use of APC based on the evidence that is currently available.

#### Formalin treatment (recommendation strength: B; level of evidence: 2a) [2, 6, 7, 9]

Four guidelines [2, 6, 7, 9] mentioned or supported topical application of formalin, and two guidelines [8, 9] did not. One guideline [6] referred to six studies with 182 cases, most of which improved with no bleeding. Guideline [9] referred to two randomized controlled trials [17, 18]. The first trial [17] involved 102 patients. Local application of formalin and ammonium thioglycolate retention enemas showed 90% and 74.5% effective rates, respectively. Another study [18] was not very supportive, comparing colonic lavage with antibiotic administration and a local application of 4% formalin. A total of 50 participants were studied; 20 in the flushing group improved, 10 in the formalin group improved, and the effect in the formalin group was slightly worse. A guideline [7] referred to the use of local formalin in multiple case reports with poor prognosis. Formalin surgery is similarly ineffective. The systematic review analysis mentioned in guideline [2] showed that local application of formalin had a good effect, and the response rate was as high as 80–100%. However, there were a series of complications, such as severe pain, colitis, perforation, stenosis, ulcers and anal incontinence. Therefore, local treatment with formaldehyde should be cautiously employed [2].

#### Bipolar electrocoagulation (recommendation strength: A; level of evidence: 1b) [2, 6, 9]

Three guidelines [2, 6, 9] mentioned or supported bipolar electrocoagulation, and three did not [7, 8, 11]. Two guidelines [2, 9] referred to four studies [13, 19–21], three randomized controlled studies and one case report involving 96 patients, with an overall success rate of 88% (95% confidence interval, moderate heterogeneity), two of which compared argon plasma [13] with heater probes [19]. The results [19] showed that bipolar coagulation is as effective as argon plasma and heater probes. At present, there are no reports of perforation or fistula formation after bipolar electrocoagulation [9]. As mentioned in study [13], the efficacy of argon plasma is similar to that of bipolar electrocoagulation. It seems that argon plasma is relatively safe, and the incidence of complications of bipolar electrocoagulation needs to be evaluated by a larger study. Based on the above evidence, bipolar electrocoagulation is not currently recommended.

#### Heater probe (recommendation strength: A; level of evidence: 1b)[2, 9]

Two of the included guidelines referred to heater probes [2, 9], and the remaining four did not. Guideline [9] referred to two studies, one randomized controlled trial [20] involving 9 patients that compared bipolar electrocoagulation with heater probes, with a clinical response rate of 67%. The other study [22] was a case report with a total of 8 patients and a clinical response rate of 100%. Guideline [2] mentioned that the hemostatic effect of the heater probe was comparable to that of bipolar electrocoagulation. Available experimental data are too limited to support its use.

#### Radiofrequency ablation (RFA) (recommendation strength: C; level of evidence: 4) [9]

Only one guideline [9] referred to radiofrequency ablation, and there were three case reports [23–25] involving 66 patients, with an overall success rate of 97.0% and bleeding improvement. The study had a small sample size and unknown safety, so RFA is not yet recommended.

#### Cryoablation (recommendation strength: C; level of evidence: 4) [9]

Cryoablation was mentioned in only one guideline [9], which referred to two case reports involving 10 [26] and 7 [27] patients with 70% and 100% response rates, respectively, and an adverse reaction (perforation) rate of 10%. Both cryoablation systems used in the study were discontinued. No data have been published on the treatment of chronic proctitis with a new generation of cryoablation systems. There is insufficient evidence to support or oppose the use of a new generation of cryoablation systems for the treatment of chronic radiation-induced rectal bleeding in patients with chronic radiation-induced rectal disease.

This study provides some suggestions for the future diagnosis and treatment of radioactive enteritis: (I) Guideline authors should use systematic retrieval methods for evidence retrieval when writing guidelines, and display the diagnosis and treatment methods of radioactive enteritis as comprehensively as possible, instead of being limited to a certain classification of radioactive enteritis, to facilitate the reference of the users of the guideline. (II) If conditions permit, experiments with larger sample sizes should be carried out to improve quality. (III) Any recommendation should provide a detailed source of evidence so that guide users can review it at their discretion and decide whether to use the guidelines or not. (IV) Guide writers should be familiar with guide evaluation tools, such as the AGREE II tool. (V) The tool manual should be provided for users to view quickly. (VI) Taking into account the opinions of the people to whom the guidelines are applied will go a long way in improving the quality of the guidelines. (VII) Inviting a third party to review the guide will greatly improve its reliability. (VIII) More consideration should be given to the hindrance and facilitation factors of guidelines in the writing process. (IX) Rather than treating radiation proctitis and enteritis, it is better to use appropriate modern radiotherapy techniques to minimize the radiation dose to the rectum and intestines, such as intensity regulation and rectal septal hydrogel [28]. These measures to prevent radiation enteritis should also be included in the diagnostic and therapeutic guidelines for radiation enteritis.

### Strengths and limitations

Every study has its advantages and limitations, and our study is no exception. The strengths of our study are as follows: (I) we attempted to review guidelines and recommendations independently and objectively; (II) to make it as convenient as possible for users, such as clinicians, to make a better choice of treatment options, we listed various recommendations; and (III) as much as possible, the retrieved literature was collated and compared to provide an improvement direction for guideline makers in the future. There are also some limitations in our study: (I) the languages used in the literature included only English and Chinese, which cannot fully represent global research results; (II) the guideline evaluation tool we used can only evaluate the guideline formulation method and cannot represent the therapeutic effect of the proposed recommendation itself, and the scoring is somewhat subjective; (III) the selection of retrieval words may have led to incomplete retrieval; (IV) clinical success was broadly defined as 10% improvement or normalization of hemorrhage- stopping hemoglobin in accordance with guidelines; and [9] bleeding score improvement or telangiectasis eradication and radioactive enteritis has a variety of classifications and a variety of different forms of expression, and cannot include all types, only the evaluation of blood type, and there are certain limitations.

## Conclusion

This study found that the differences in the methodological quality of the guidelines for radioactive enteritis, even within the same guideline, were pronounced between different domains, especially in the scope and purpose of stakeholder rigor. When applied, there was much dissent, and the opinions varied. The high consistency of the recommendations was due to the argon ion coagulation technique (argon local use of 4% or 10% formalin has not been proven to have better efficacy). Other treatments, such as radiofrequency ablation and cryoablation, also need more experimental data to prove their safety and efficacy. It is hoped that these observations will be taken into account when new guidelines are developed.

## Supplementary Information

**Additional file1**. **Table S1**: Levels of evidence and grades of the recommendations based on the Oxford Centre for Evidence-Based Medicine.

## Data Availability

All authors agree to share the data of this review, which can be obtained by contacting the corresponding authors. Email: sundali2018@126.com.

## Abbreviations

AGREE II: The Appraisal of Guidelines for Research and Evaluation II
ICC: Intra-class correlation coefficient
ASGE: American Society for Gastronintestinal Endoscopy
APC: Argon plasma coagulation
RFA: Radiofrequency ablation
OVID: Ovid Technologies
CBM: China Biology Medicine Disc
CNKI: China National Knowledge Internet
R: Recommended
RM: Recommended with modifications
NR: Not recommended
SPSS: Statistic Package for Social Science
OCEBM: Oxford Centre for Evidence-Based Medicine
RCT: Randomized controlled trial
GRADE: Grading of Recommendations Assessment Development and Evaluation
USA: The United States of America
SR: Systematic review
ASGE: American Society for Gastrointestinal Endoscopy
CMA: Chinese Medical Association
