# Supplementary material for: Quality evaluation of guidelines for the diagnosis and treatment of radiation enteritis
Source: Radiat Oncol. 2023 Jan 20;18:14. doi: 10.1186/s13014-023-02204-9 (PMC9862547; doi:10.1186/s13014-023-02204-9)
Supplement: Supplementary file 1 — Additional file1. Table S1: Levels of evidence and grades of the recommendations based on the Oxford Centre for Evidence-Based Medicine. [file 13014_2023_2204_MOESM1_ESM.docx]

Table S1 Levels of evidence and grades of the recommendations based on the Oxford Centre for Evidence-Based Medicine

| Strength of  recommendation | Quality  of evidence | Description |
| --- | --- | --- |
| A | 1a  1b | Systematic review (SR) with homogeneity of randomized controlled trials (RCTs)  Individual RCT (with narrow confidence interval) |
|  | 1c | All or none |
| B | 2a | SR (with homogeneity) of cohort studies |
|  | 2b | Individual cohort study (including low-quality RCT; for example, <80% follow-up) |
|  | 2c | “Outcomes” research; ecological studies |
|  | 3a | SR with homogeneity of case-control studies |
|  | 3b | Individual case-control study |
| C | 4 | Case series (and poor-quality cohort and case-control studies) |
| D | 5 | Expert opinion without explicit critical appraisal, or based on physiology, bench research, or  “first principles” |
